# Single-cell RNA-seq and spatial transcriptomics characterize CD8^+^ exhausted T cells in pancreatic ductal adenocarcinoma

**DOI:** 10.1016/j.isci.2026.116934

**Published:** 2026-07-27

**Authors:** Jing Mao, Chenxin Yan, Ying Mei, Yanjun Yao, Xingxia Yang, Jing Zhuang, Kuai Yu, Gangzhao Gu, Hengzhi Zhang, Yu Zheng, Yunyao Wei, Shuwen Han, Qiang Yan

**Affiliations:** 1 Huzhou Central Hospital, Affiliated Huzhou Hospital, Zhejiang University School of Medicine, Huzhou, Zhejiang, China; 2 The Second Affiliated Hospital of Nanchang University, Nanchang, Jiangxi 330006, China; 3 Shulan International Medical College, Zhejiang Shuren University, Hangzhou, Zhejiang, China; 4 Huzhou Central Hospital, Affiliated Central Hospital Huzhou University, Huzhou, Zhejiang, China; 5 Huzhou Central Hospital, Fifth School of Clinical Medicine of Zhejiang Chinese Medical University, Huzhou, Zhejiang, China; 6 Lishui Central Hospital, Lishui, Zhejiang, China; 7 Jiaxing University Affiliated Women and Children Hospital, Jiaxing, Zhejiang, China; 8 School of Pharmacy, Hangzhou Normal University, Hangzhou, Zhejiang, China; 9 ASIR (Institute - Association of Intelligent Systems and Robotics), Rueil-Malmaison, France; 10 Boron Neutron Capture Cancer Treatment Technology, Zhejiang Engineering Research Center, Huzhou, Zhejiang, China; 11 Huzhou Key Laboratory of Intelligent and Digital Precision Surgery, Huzhou, Zhejiang, China

**Keywords:** pancreatic ductal adenocarcinoma, single-cell RNA sequencing, spatial transcriptome, CD8^+^exhausted T cell

## Abstract

Pancreatic cancer resists immunotherapy due to a suppressive immune microenvironment where CD8^+^ T cells play a key role. Using single-cell RNA sequencing and spatial transcriptomics, we characterized CD8^+^ exhausted T (Tex) cells in pancreatic ductal adenocarcinoma (PDAC). We generated single-cell profiles from PDAC tumors and matched peripheral blood mononuclear cells, and performed T cell sub-analysis. We found CXCL13 upregulated and GZMK downregulated in CD8^+^ Tex cells. Cell-cell interaction analysis showed that T cells most frequently interacted with myeloid cells and cancer cells via ligand-receptor pairs; INHBA^+^ macrophages and cancer cells communicated most with CD8^+^ Tex cells. Two key LR pairs (SPP1-integrin α4β1 and PLAUR-integrin α4β1) mediated crosstalk between cancer cells and CD8^+^ Tex cells, confirmed by immunofluorescence, spatial mapping, and protein docking. High SPP1 and PLAUR expression correlated with poor prognosis in TCGA-PAAD. These findings provide a resource for understanding CD8^+^ T cell exhaustion in PDAC.

## Highlights

• Single-cell and spatial transcriptomics map CD8^+^T cell exhaustion in PDAC
• SPP1^+^/PLAUR^+^cancer cells and INHBA^+^macrophages interact with exhausted CD8^+^T cells
• SPP1/α4β1 and PLAUR/α4β1 are key ligand-receptor pairs mediating cancer-T cell crosstalk
• High SPP1 and PLAUR expression correlate with poor prognosis in PDAC

## Graphical abstract

**Figure:**
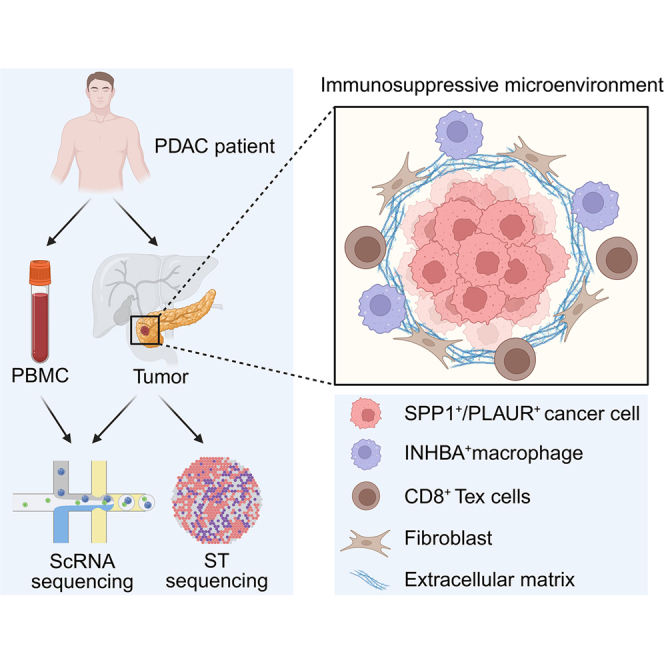

## Teaser

Medicine; Oncology; Cancer

## Introduction

Pancreatic ductal adenocarcinoma (PDAC) constitutes over 90% of pancreatic cancer cases and remains one of the most lethal malignancies worldwide, with rising incidence and persistently low survival rates.^1^^,^^2^ The latest global cancer statistics show that pancreatic cancer is the sixth leading cause of cancer death, accounting for 4.8% of all cancer deaths.^3^ According to the data released by the American Cancer Society, the 5-year survival rate of pancreatic cancer has increased from 3% to 4% 20 years ago to 13% at present, but its survival rate is still the lowest among all malignant tumors.^4^^,^^5^ The early stage of pancreatic cancer often lacks typical clinical symptoms, and most patients clinically diagnosed with pancreatic cancer have entered the middle and advanced stage. Surgical resection is currently the most effective treatment for pancreatic cancer, but only 20% of pancreatic cancer patients have the opportunity to undergo surgery.^6^ Patients who underwent surgical resection are also prone to recurrence and metastasis after surgery, and their 5-year survival rate is only 15%–20%.^2^^,^^7^ Chemotherapy remains the standard treatment for advanced PDAC, but drug resistance frequently develops, and overall survival seldom exceeds two years.^8^ In recent years, immunotherapy has brought new vitality to tumor patients and achieved curative effect in a variety of solid tumors. However, its clinical application effect in pancreatic cancer has not met expectations. This lack of response is largely attributed to the highly immunosuppressive tumor microenvironment characteristic of PDAC.^9^ Its specificity is mainly manifested in the following aspects: (1) a small amount of infiltration of effector lymphocytes and a majority of effector lymphocyte dysfunction in pancreatic cancer, such as reduced infiltration and exhaustion of CD8^+^T cells, leading to inactivation of anti-tumor immunity^10^; (2) the microenvironment of pancreatic cancer contains a variety of immunosuppressive cells, including tumor-associated macrophages and myeloid-derived suppressor cells^11^^,^^12^; (3) the proliferation of fibrous connective tissue and the large number of stromal components make it difficult for drugs to penetrate into the tumor and create an immunosuppressive microenvironment^13^; and (4) immunosuppressive factors are increased in the pancreatic cancer microenvironment, interfering with the body’s normal immune response to cancer cells.^14^^,^^15^ CD8^+^exhausted T (Tex) cells are one of the most important factors.

CD8^+^Tex cells are a key component of the tumor immune microenvironment and contribute significantly to tumor immune escape, which underlies the poor response rates to immunotherapy.^16^ CD8^+^T cells exhaustion is accompanied by the expression of some of their coinhibitory receptors, including PD-1, and thus the use of immune checkpoint blockers is expected to reactivate these PD-1-expressing exhausted cells from a nonfunctional state. With the development and advancement of methods such as single-cell RNA sequencing (scRNA-seq), the understanding of CD8^+^Tex cells is being transformed. Factors such as the heterogeneity and spatial distribution of CD8^+^Tex cells may also influence their response to immune checkpoint blockers.^17^^,^^18^ However, the reasons that affect the exhausted CD8^+^T cell state in the pancreatic cancer microenvironment are not clear. Therefore, characterizing the heterogeneity and spatial architecture of CD8^+^Tex cells in PDAC could provide a foundation for elucidating mechanisms of immunotherapy resistance and developing novel therapeutic approaches.

ScRNA-seq enables unbiased profiling of gene expression at single-cell resolution, overcoming the limitations of bulk sequencing and revealing cellular heterogeneity.^19^ In the immune microenvironment, scRNA-seq can provide in-depth analysis of the state and function of different immune cells and reveal their mechanism of action in the occurrence and development of diseases, thereby providing new targets and strategies for disease diagnosis and treatment. Spatial transcriptomics (ST) complements scRNA-seq by mapping gene expression back to tissue architecture, providing insights into cellular interactions and spatial expression patterns.^20^ However, both of these two technologies have certain defects. The results of scRNA-seq lack spatial location information, and the accuracy of spatial transcriptome sequencing cannot reach the level of single-cell detection. Such combined strategies have recently been applied to study cancer-associated fibroblasts, hypoxic niches, and precancerous lesions in PDAC, yielding valuable insights.^21^^,^^22^^,^^23^ However, an integrative and comprehensive interpretation and analysis of CD8^+^Tex cells in pancreatic cancer has not yet been performed.

To elucidate the relationship between cancer cells and CD8^+^Tex cells in the tumor microenvironment and to analyze the mechanism of exhaustion. Deep sequencing of pancreatic tissue was performed. In this study, scRNA-seq was performed on five fresh PDAC tumor tissue samples and five matched peripheral blood samples, and spatial transcriptome sequencing was performed on five formalin-fixed paraffin-embedded (FFPE) sections of PDAC tumor tissue from the same five patients, enabling integrated analysis of the same tumors. Our findings reveal the heterogeneity and communication pathway of CD8^+^Tex cells in the PDAC tumor microenvironment and peripheral blood mononuclear cells (PBMCs) of patients. It is expected to provide new targets and strategies for the comprehensive treatment of pancreatic cancer.

## Results

### Cell atlas of PDAC immune microenvironment

**Figure 1:**
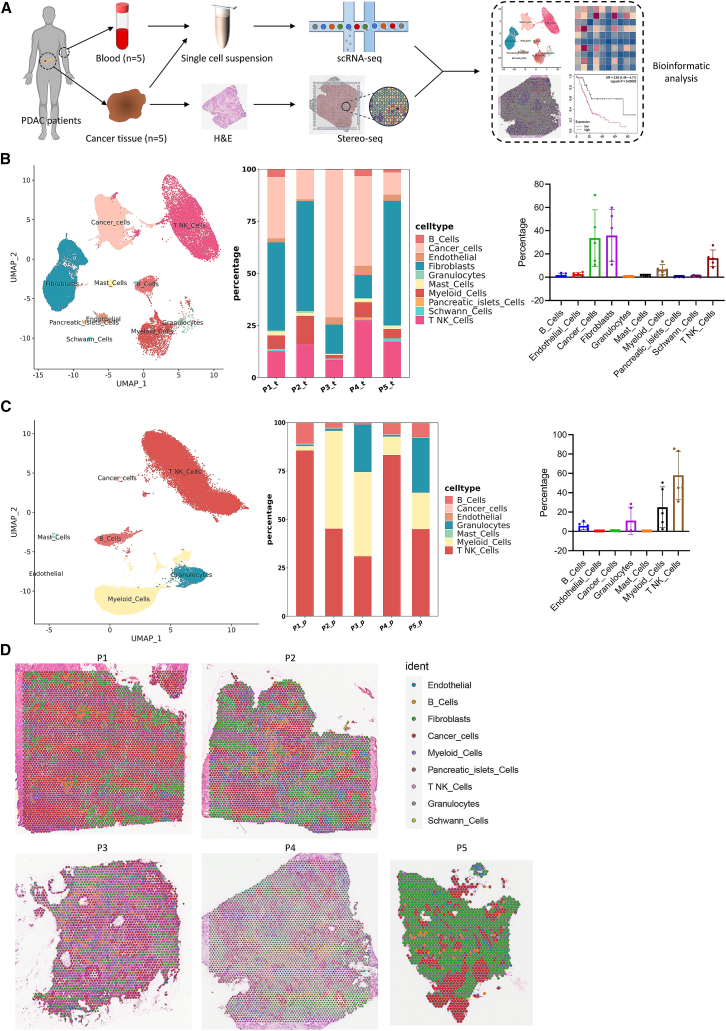
Diverse cell types in PDAC delineated by scRNA-seq and ST analysis (A) Graphic overview of this study design. Tumor tissue and peripheral blood samples from five PDAC patients were processed into single cell suspensions, and sorted cells were used for scRNA-seq by 10× Genomics. Tumor sections were processed with the Visium Spatial platform to obtain ST. (B) UMAP maps of 39,854 cells of tumor tissue from five PDAC patients, showing ten clusters (left). Each cluster is represented by a different color. Bar graph showing the proportion of ten cell types in the tumor tissue of each patient (middle). Bar diagram showing the average proportion of ten cell types in the tumor tissue (right). (C) UMAP maps of 38,632 cells of PBMC from five PDAC patients, showing seven clusters (left). Different colors represent different cell types. Bar graph showing the proportion of seven cell types in the PBMC of each patient (middle). Bar diagram showing the average proportion of seven cell types in the PBMC (right). (D) Spatial maps showing the spatial location of ten cell types in the tumor tissue on the pathological section of each patient, each of which is indicated by different colors.

We collected postoperative tumor samples and matched PBMC samples from five non-metastatic patients with pathologically diagnosed PDAC (Table S2). FASC sorting was performed on each sample cell suspension to remove dead cells and scRNA-seq analysis was performed using the 10× Genomics platform (Figure 1A). Data were screened using quality control parameters described in the R package Seurat. A total of 39,854 cellular transcriptomes from tumor tissue from five patients were retained for subsequent analyses (Table S3). The cells in the tumor tissue were divided into ten major types: B cells, cancer cells, endothelial cells, fibroblasts, granulocytes, mast cells, myeloid cells, pancreatic islet cells, Schwann cells, and T/natural killer (NK) cells. Cancer cells and fibroblasts were predominant, but the level of infiltration of these major cell types varied among samples (Figure 1B). A total of 38,632 cellular transcriptomes from PBMCs of the five patients were collected for subsequent analyses (Table S3). These cells were divided into seven major cell types (B cells, cancer cells, endothelial cells, granulocytes, mast cells, myeloid cells, and T/NK cells), with T/NK cells and myeloid cells being the dominant cells (Figure 1C). Spatial mapping of each class of cells was then performed on each of the five idled sections of tumor tissue, and the proportions of each class of cells were consistent with the scRNA-seq analysis (Figure 1D).

### Cellular biomarkers in PDAC

**Figure 2:**
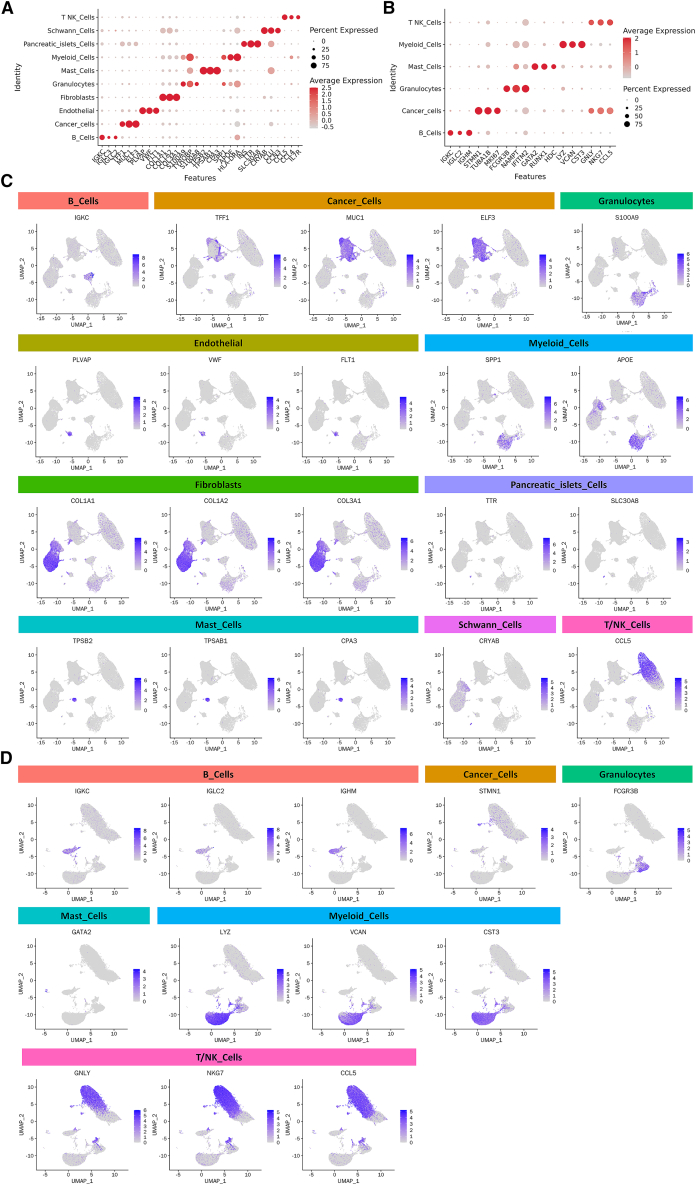
Molecular markers of various cells in PDAC tumor tissues and PBMC (A) Dot plots showing the average expression of molecular markers in the indicated cell clusters of tumor tissues. The size of the dots represents the percent of cells expressing the genes in each cluster. The intensity of marker expression is shown according to the depth of color. (B) Dot plots showing the average expression of molecular markers in the indicated cell clusters from PBMC. The size of the dots represents the percent of cells expressing the genes in each cluster. The intensity of marker expression is shown according to the depth of color. (C) Expression levels of the selected marker genes across 39,854 cells in UMAP maps from tumor tissues in PDAC patients. (D) Expression levels of the selected marker genes across 38,632 cells in UMAP plots from PBMC in PDAC patients.

Signature genes were screened in PDAC tissues and matched PBMC. Cells in the tumor tissue included B cells labeled by IGKC (*n* = 724), cancer cells expressing TFF1, MUC1, and ELF3 (*n* = 13,123), endothelial cells defined by PLVAP, VWF, and FLT1 (*n* = 1,077), Fibroblasts positive for COL1A1, COL1A2, and COL3A1 (*n* = 14,355), S100A9-labeled granulocytes (*n* = 159), and mast cells identified for TPSB2, TPSAB1, and CPA3 (*n* = 600), SPP1 and APOE-labeled myeloid cells (*n* = 2,771), TTR and SLC30A8 labeled pancreatic islet cells (*n* = 134), CRYAB-expressing Schwann cells (*n* = 257), and CCL5-labeled T/NK cells (*n* = 6,654) (Figures 2A and 2C). The top ten differentially expressed marker genes in various types of cells can be viewed in Table S4. Cells in PBMC included B cells labeled by IGKC, IGLC2, and IGHM (*n* = 2,268), cancer cells expressing STMN1 (*n* = 130), granulocytes labeled by FCGR3B (*n* = 3,107), mast cells expressing GATA2 (*n* = 28), Myeloid cells labeled with LYZ, VCAN, and CST3 (*n* = 9,219) and T/NK cells labeled with GNLY, NKG7, and CCL5 (*n* = 23,878) (Figures 2B and 2D). The top ten differentially expressed marker genes in various types of cells are shown in Table S5.

### Composition of infiltrating and circulating T cells in PDAC

**Figure 3:**
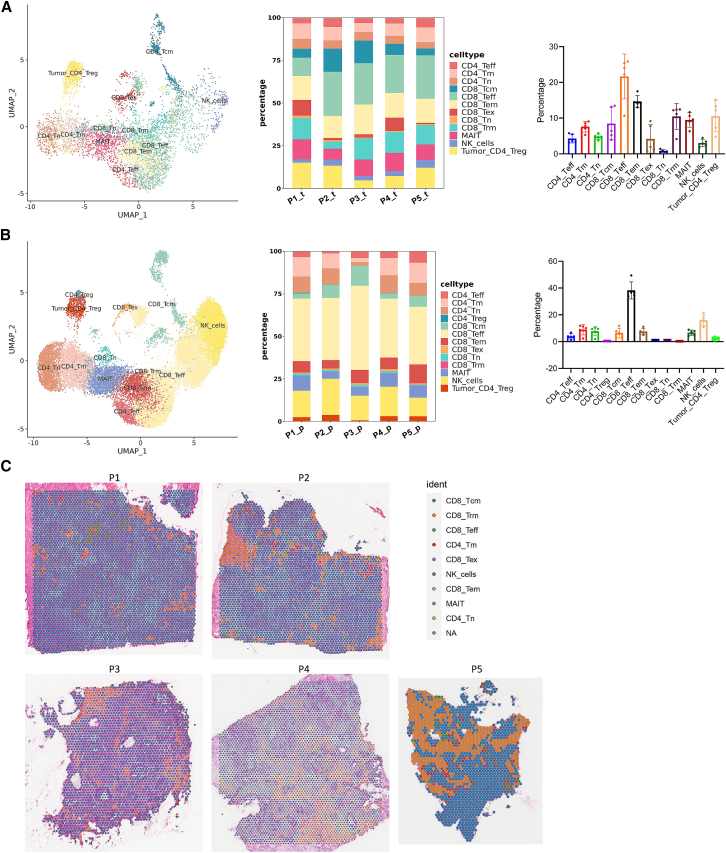
Infiltrating and circulating T cell types in PDAC analyzed by scRNA-seq and ST (A) UMAP maps of 6,715 T cells of tumor tissue from five PDAC patients, showing 12 clusters (left). Each cluster is represented by a different color. Bar graph showing the proportion of 12 cell types in the tumor tissue of each patient (middle). Bar diagram showing the average proportion of 12 T cell types in the tumor tissue (right). (B) UMAP maps of 24,427 T cells of PBMC from five PDAC patients, showing 13 clusters (left). Different colors represent different cell types. Bar graph showing the proportion of 13 cell types in the PBMC of each patient (middle). Bar diagram showing the average proportion of 13 T cell types in the PBMC (right). (C) Spatial maps showing the spatial location of 12 cell types in the tumor tissue on the pathological section of each patient, each of which is indicated by different colors.

We further analyzed the T cell composition in tumor tissue and divided 6,715 T cells (Table S6) into 12 clusters (CD4^+^effector T [Teff] cells, CD4^+^memory T [Tm] cells, CD4^+^naive T [Tn] cells, CD8^+^central memory T [Tcm] cells, CD8^+^Teff cells, CD8^+^effector memory T [Tem] cells, CD8^+^exhausted T [Tex] cells, CD8^+^Tn cells, CD8^++^tissue-resident memory T [Trm] cells, mucosal-associated invariant T [MAIT] cells, NK cells, CD4^+^T regulatory [Treg] cells) among which CD8^+^Teff cells and CD8^+^Tem cells were predominant (Figure 3A). In the analysis of the T cell composition of the patient’s PBMC, we divided the 24,429 T cells (Table S6) into 13 subclasses (CD4^+^Teff cells, CD4^+^Tm cells, CD4^+^Tn cells, CD4^+^Treg cells, CD8^+^Tcm cells, CD8^+^Teff cells, CD8^+^Tem cells, CD8^+^Tex cells, CD8^+^Tn cells, CD8^+^Trm cells, MAIT cells, NK cells, tumor CD4^+^Treg cells). Among them, CD8^+^Teff cells and NK cells were predominant, and they were significantly higher than those in tumor tissues (Figure 3B). The spatial distribution of various T cell subsets was mapped on five tissue-invariant sections, all of which showed a high prevalence of CD8^+^Tcm cells (Figure 3C).

### Molecular markers of infiltrating and circulating T cells in PDAC

**Figure 4:**
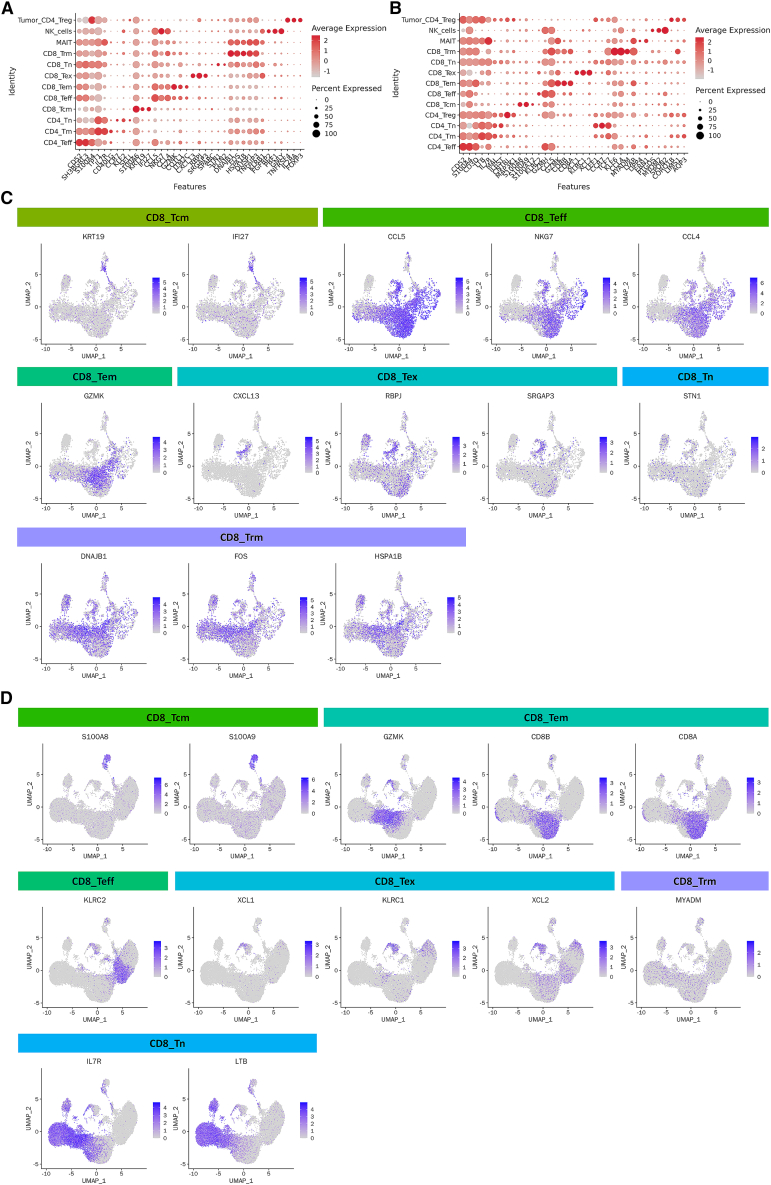
Molecular markers of infiltrating and circulating T cells in PDAC tumor tissues and PBMC (A) Dot plots showing the average expression of molecular markers in the indicated T cell clusters of tumor tissues. The size of the dots represents the percent of T cells expressing the genes in each cluster. The intensity of marker expression is shown according to the depth of color. (B) Dot plots showing the average expression of molecular markers in the indicated T cell clusters from PBMC. The size of the dots represents the percent of T cells expressing the genes in each cluster. The intensity of marker expression is shown according to the depth of color. (C) Expression levels of the selected marker genes across 6,715 T cells in UMAP maps from tumor tissues in PDAC patients. (D) Expression levels of the selected marker genes across 24,427 T cells in UMAP plots from PBMC in PDAC patients.

We screened for signature genes of T cells in PDAC tumor tissues and matched PBMC. In tumor tissues, these T cell subclasses included CD52-tagged CD4^+^Teff cells (*n* = 293), CD4^+^Tm cells defined by TPT1 and IL7R (*n* = 515), CCR7 and KLF2-positive CD4^+^Tn cells (*n* = 317), CD8^+^Tcm cells labeled with KRT19 and IFI27 (*n* = 537), CD8^+^Teff cells identified by CCL5, NKG7, and CCL4 expression (*n* = 1,496), CD8^+^Tem cells expressing GZMK (*n* = 962), CD8^+^Tex cells labeled by CXCL13, RBPJ and SRGAP3 (*n* = 309), CD8^+^Tn cells expressing STN1 (*n* = 51), CD8^+^Trm cells defined by DNAJB1, FOS and HSPA1B (*n* = 689), TNFAIP3 and KLRB1 labeled MAIT (*n* = 632), FGFBP2 and PRF1 labeled NK cells (*n* = 212), expressed TNFRSF4, IL2RA, and FOXP3 tumor CD4^+^Treg cells (*n* = 702) (Figure S1A; Figures 4A–4C). We recorded the top ten differentially expressed marker genes in the above types of cells in Table S7. In PBMC, these T cell subclasses included CD52-tagged CD4^+^Teff cells (*n* = 895), CD4^+^Tm cells defined by LTB and IL7R (*n* = 2,411), CCR7-positive CD4^+^Tn cells (*n* = 2,212), MAP3K1-positive CD4^+^Treg cells (*n* = 55), CD8^+^Tcm cells labeled by S100A8 and S100A9 (*n* = 1,198), CD8^+^Teff cells identified by KLRC2 expression (*n* = 8,964), CD8^+^Tem cells expressing GZMK, CD8B and CD8A (*n* = 1,704), CD8^+^Tcm cells labeled by S100A8 and S100A9 (*n* = 1,198), CD8^+^Teff cells identified by KLRC2 expression (*n* = 8,964), CD8^+^TEM cells expressing GZMK, CD8B and CD8A (*n* = 1,704). CD8^+^Tex cells labeled by XCL1, KLRC1, and XCL2 (*n* = 228), CD8^+^Tn cells expressing IL7R and LTB (*n* = 231), CD8^+^Trm cells defined by MYADM (*n* = 3), MAIT labeled by GZMK, IL7R and LTB (*n* = 1,775), PTGDS, MYOM2, and SPON2 labeled NK cells (*n* = 4,064) expressing LTB and RTKN2 tumor CD4^+^Treg cells (*n* = 689) (Figure S1A; Figures 4B–4D). The top ten differentially expressed genes in these 13 classes of T cells are shown in Table S8.

### Molecular characteristics of CD8^+^Tex cells in PDAC

**Figure 5:**
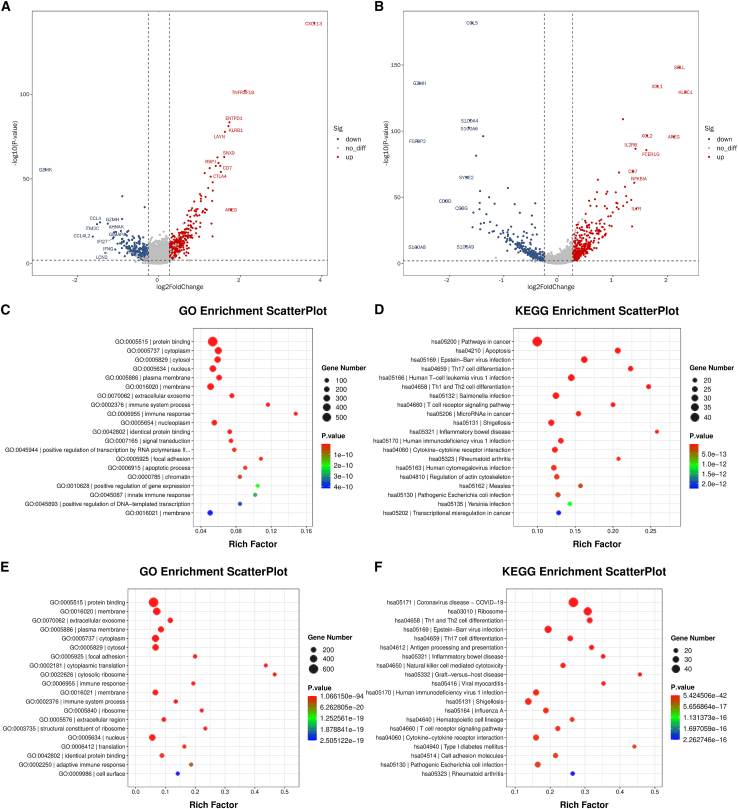
Comparison of CD8^+^Tex cells with CD8^+^non-Tex cells in PDAC tumor tissues and PBMC (A) Volcano plot showing DEGs between CD8^+^Tex cells and CD8^+^non-Tex cells in PDAC tumor tissues. Red dots represent genes that are up-regulated in CD8^+^Tex cells, and blue dots represent genes that are down-regulated in CD8^+^Tex cells. (B) Volcano plot showing DEGs between CD8^+^Tex cells and CD8^+^non-Tex cells in PBMC. Red dots represent genes that are up-regulated in CD8^+^Tex cells, and blue dots represent genes that are down-regulated in CD8^+^Tex cells. (C) Bubble plots showing GO enrichment analysis of DEGs in tumor. (D) Bubble plots showing KEGG enrichment analysis of DEGs in tumor. (E) Bubble plots showing GO enrichment analysis of DEGs in PBMC. (F) Bubble plots showing KEGG enrichment analysis of DEGs in PBMC.

To understand the molecular characteristics of CD8^+^Tex cells in the tumor microenvironment of PDAC, we compared the difference between CD8^+^Tex cells and CD8^+^ non-Tex cells in tumor tissues and PBMC, respectively. In tumor tissues, a total of 693 DEGs were screened between the two groups, of which 456 were up-regulated and 237 were down-regulated (Figure 5A). The top 20 DEGs are shown in Table S9. In PBMC, a total of 793 differentially expressed genes were identified, of which 425 were up-regulated and 368 were down-regulated (Figure 5B). The top 20 differentially expressed genes are listed in Table S10. Comparison of DEGs between CD8^+^Tex cells in tumor and PBMC revealed both shared and unique genes. TNFRSF18, AREG, KLRB1, CD7, and XCL1 were upregulated in Tex cells from both compartments, confirming its role as a universal exhaustion marker. GZMK was downregulated in CD8^+^Tex cells of the tumor microenvironment, but upregulated in CD8^+^Tex cells of PBMCs. This completely opposite phenomenon indicates that there might be other inhibitory signals in the tumor microenvironment that further suppressed the activity of the effector molecules. To investigate the functions and possible signaling pathways of DEGs, we performed GO and KEGG enrichment analysis of DEGs. In tumor tissues, these DEGs were related to exosome signaling, immune response process, apoptosis, innate immune response, and other functions, and were mostly enriched in T cell differentiation (such as Th1, Th2, and Th17), cytokine-cytokine receptor interaction and other signaling pathways (Figures 5C and 5D). The DEGs in PBMC also showed similar results, which were related to exosome signaling, immune response process, adaptive immune response and other functions. It was enriched in signaling pathways such as T cell differentiation (such as Th1, Th2, and Th17), NK cell-mediated cytotoxicity, and cytokine-cytokine receptor interaction (Figures 5E and 5F).

### LR analysis between CD8^+^Tex cells and macrophages in PDAC

**Figure 6:**
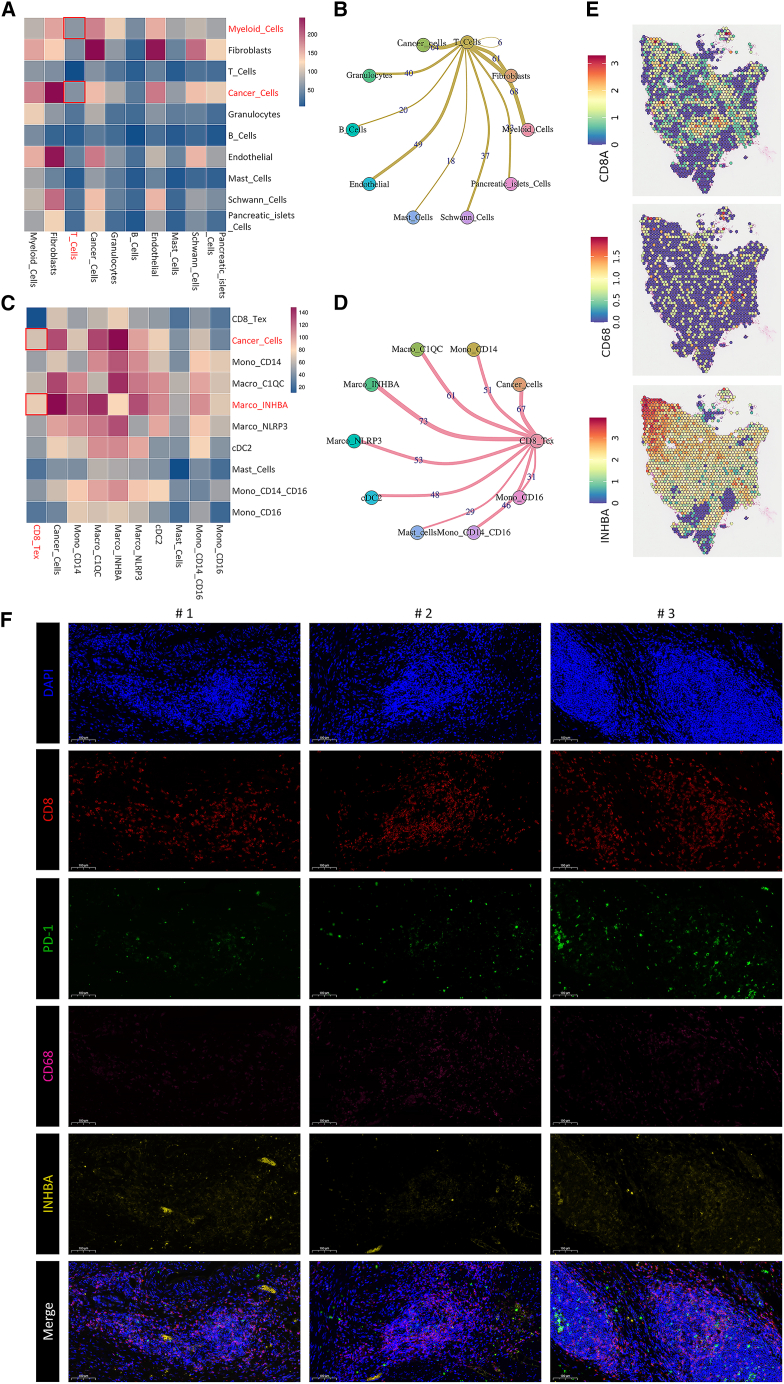
LR interactions between CD8^+^Tex cells and macrophages in PDAC (A) Heatmap showing the number of LR pairs between ten types of cells in PDAC tumor tissue. (B) Interaction network diagram showing the specific number of LR pairs between T cells and the remaining nine types of cells in PDAC tumor tissues. (C) Heat maps showing the number of LR pairs between CD8^+^Tex cells, cancer cells, and eight types of myeloid cells in PDAC tumor tissues. (D) Interaction network diagram showing the specific number of LR pairs between CD8^+^Tex cells and cancer cells, eight types of myeloid cells in PDAC tumor tissues. (E) Spatial maps showing the distribution of CD8A, CD68, and INHBA in pathological sections of tumor tissues from P5 patient. Different colors represent different expression levels of genes. (F) Multiplex immunofluorescence staining of human PDAC tumor tissue (20×). CK (cyan), DAPI (blue), CD8 (red), PD-1 (green), CD68 (pink), and INHBA (orange), in individual and merged channels are shown. Bars, 50 μm. The experiments were performed in five patients.

ScRNA-seq data were used to predict potential LR interactions among various types of cells in the PDAC microenvironment. These interactions revealed the underlying mechanisms leading to PDAC immunosuppression. We applied quantitative predictions based on LR relation pairs to assess potential relationships between major cell types. Heat maps of interactions showed that T cells and myeloid cells exchanged the most information (Figures 6A and 6B). Exhaustion of CD8^+^T cells is one of the important causes of immune suppression in the microenvironment of PDAC. Therefore, we performed further cell subpopulation LR interactions analysis of T cells and myeloid cells. We found that CD8^+^Tex cells communicated most frequently with INHBA^+^macrophages (Figures 6C and 6D). Spatial mapping of sections and multiplex immunofluorescence staining were used to verify the relationship between the two cell types, which confirmed the proximity of the two cell types in tissue space (Figures 6E and 6F).

### LR analysis between CD8^+^Tex cells and cancer cells in PDAC

**Figure 7:**
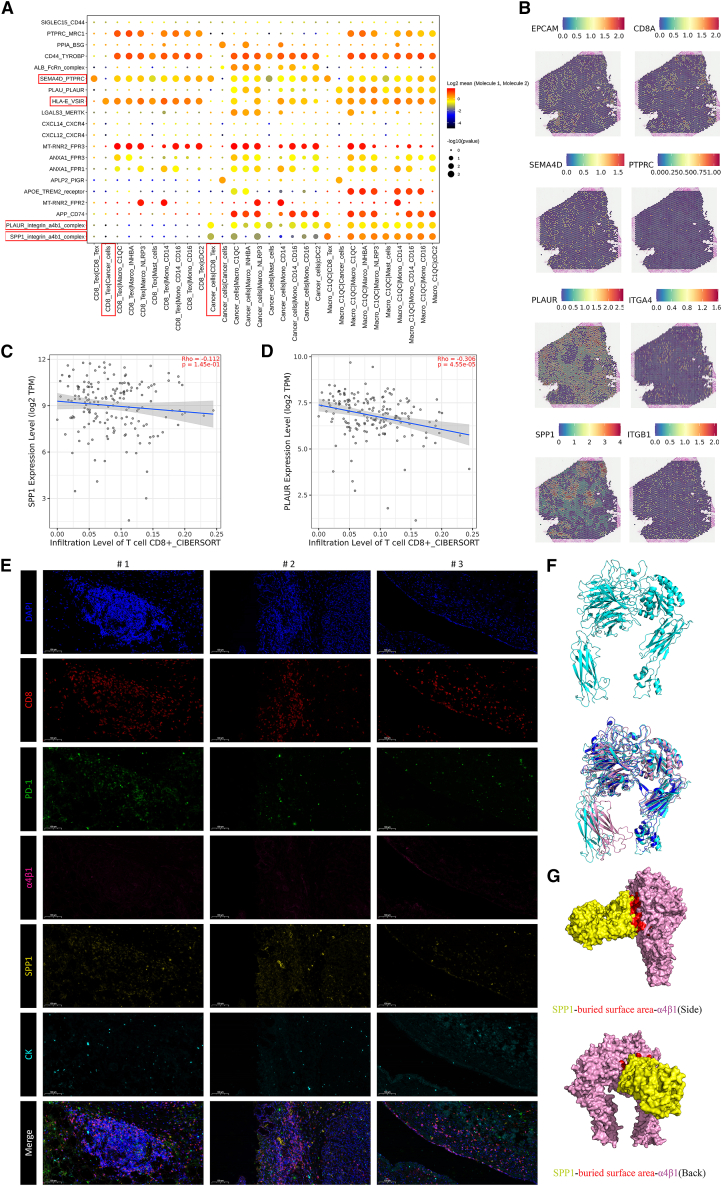
LR interactions between CD8^+^Tex cells and cancer cells in PDAC (A) Bubble heat maps showing LR pairs that interact between CD8^+^Tex cells, and cancer cells. (B) Spatial maps showing the distribution of EPCAM, SEMA4D, PLAUR, SPP1, CD8A, PTPRC, ITGA4, and ITGB1 in pathological sections of tumor tissues from P4 patient. Different colors represent different expression levels of genes. (C) Correlation analysis between the expression levels of SPP1 and CD8^+^T cell infiltration in pancreatic cancer from TIMER2.0. (D) Correlation analysis between the expression levels of PLAUR and CD8^+^T cell infiltration in pancreatic cancer from TIMER2.0. (E) Multiplex immunofluorescence staining of human PDAC tumor tissue (20×). CK (cyan), DAPI (blue), CD8 (red), PD-1 (green), α4β1 (pink), and SPP1 (orange), in individual and merged channels are shown. Bars, 100 μm. The experiments were performed in five patients. (F) Structural comparison of α4β1 integrin protein (cyan) with α4β7 (PDB ID:3V4V, pink) and α5β1 (PDB ID:4WK0, blue). (G) Visualization analysis by PyMOL showing that α4β1 (purple) and SPP1 (cyan) have good spatial binding (side and back). The binding site is shown in red.

In the prediction of LR interactions, we found that T cells or CD8^+^Tex cells were second only to myeloid cells or INHBA^+^macrophages in the degree of communication with cancer cells (Figures 6A–6D). Further analysis showed that there were three important LR pairs in the interaction between cancer cells and CD8^+^Tex cells, namely SEMA4D and PTPRC, PLAUR and α4β1, SPP1 and α4β1, while HLA-E and VSIR were important LR pairs between CD8^+^Tex cells and cancer cells (Figure 7A; Table S11). According to the corresponding cell markers, we verified the four pairs of LR in spatial mapping, among which the proximity of SPP1^+^cancer cells to α4β1^+^CD8^+^Tex cells and PLAUR^+^cancer cells to α4β1^+^CD8^+^Tex cells were the most obvious (Figure 7B). The spatial mapping relationship between HLA-E and PTPRC is not shown in the results due to the absence of probe capture for HLA-E and the low proportion of PTPRC expression. To understand the relationship between the expression of SPP1, PLAUR, and CD8^+^T cell infiltration in the tumor microenvironment, we performed single-gene immune infiltration analysis in TIMER2.0 (Figures 7C and 7D). We found a trend of negative correlation between SPP1 expression levels and CD8^+^T cell infiltration, but unfortunately a meaningful *p* value could not be obtained. PLAUR expression level was negatively correlated with CD8^+^T cell infiltration (*p* < 0.05). Multiplex immunofluorescence staining of sections showed spatial co-localization of SPP1^+^cancer cells and PLAUR^+^cancer cells with α4β1^+^CD8^+^Tex cells, respectively (Figure 7E; Figure S2A). The structure of α4β1 was predicted and verified by structural comparison with α4β7 (PDB ID:3V4V) and α5β1 (PDB ID:4WK0) (Figure 7F). In the Ramachandran plot, amino acid residues that fall in the most favored regions and the additional allowed regions account for more than 90% of the whole protein (Figure S3). Thus, the conformation of the model conforms to the rules of stereochemistry. The visualization results showed that α4β1 had a good combination with SPP1 and PLAUR (Figure 7G; Figure S2B), and the docking scores were −274.80 and −264.10, respectively.

### Expression of molecules associated with CD8^+^T cell exhaustion and their relationship with PDAC prognosis

**Figure 8:**
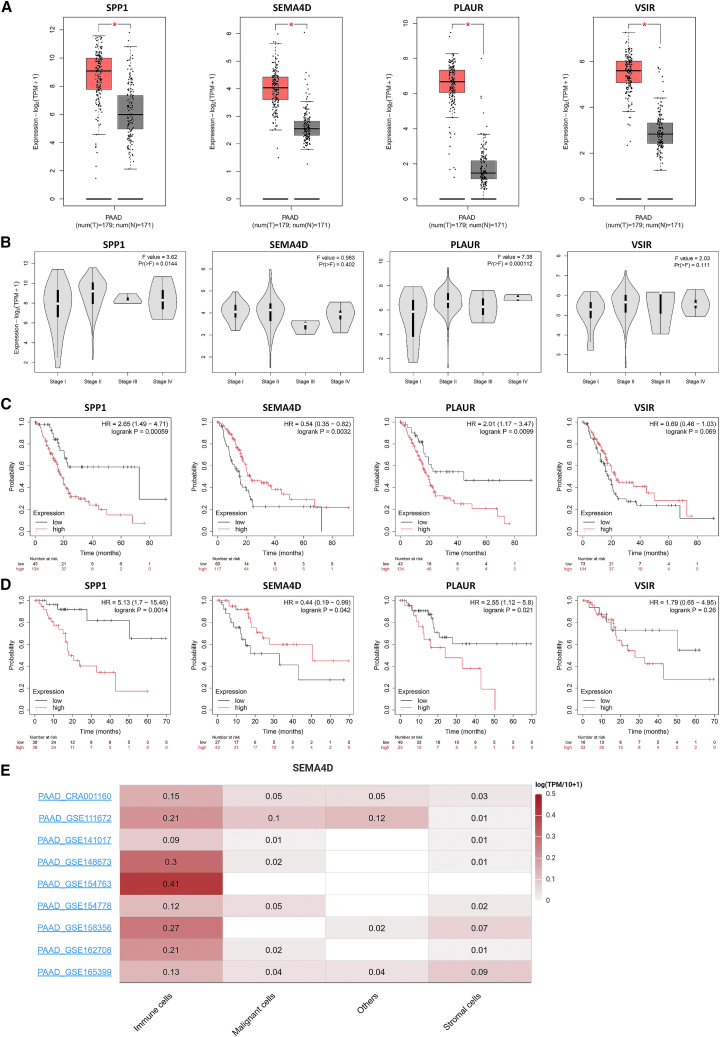
Roles of four molecules associated with CD8^+^T cell exhaustion in PDAC (A) The different expression levels of SPP1, SEMA4D, PLAUR, and VSIR in tumor and adjacent normal tissues in TCGA_PAAD and normal pancreas in GTEx (∗*p* < 0.05). (B) SPP1, SEMA4D, PLAUR, and VSIR expression among subjects with different stages in TCGA_PAAD. (C) K-M survival curve showing the relationship between the expression levels of SPP1, SEMA4D, PLAUR, VSIR and OS of patients. (D) K-M survival curve showing the relationship between the expression levels of SPP1, SEMA4D, PLAUR, VSIR, and RFS of patients. (E) Heatmap showing the expression level of SEMA4D on immune cells, malignant cells, stromal cells and other cells in CRA001160, GSE111672, GSE141017, GSE148673, GSE154763, GSE154778, GSE158356, GSE162708, GSE165399databases.

We further analyzed the relationship between the expression of SPP1, SEMA4D, PLAUR, VSIR and PDAC on the GEPIA2 website. The results showed that the expression levels of SPP1, SEMA4D, PLAUR, and VSIR in tumors were higher than those in normal tissues (*p* < 0.05) (Figure 8A). We also analyzed the expression differences of SPP1, SEMA4D, PLAUR, and VSIR in different tumor stages (Figure 8B). The higher the expression level of SPP1 and PLAUR, the more advanced the stage (*p* < 0.05). The expression level of SEMA4D was higher in early stage PDAC (stage Ⅰ and Ⅱ); however, the difference was not statistically significant (*p* > 0.05). VSIR expression was higher in advanced PDAC (stage Ⅲ and Ⅳ), but the *p* value was also greater than 0.05. The prognostic analysis of the four gene expressions in PDAC was performed on the Kaplan-Meier Plotter website (Figures 8C and 8D). High expression of SPP1 and PLAUR indicated that PDAC had worse overall survival (OS) and relapse-free survival (RFS) (*p* < 0.05). High expression of SEMA4D reflected a good outcome of PDAC (*p* < 0.05). The expression level of VSIR was not correlated with the prognosis of PDAC (*p* > 0.05). To explain the better prognosis of high expression of SEMA4D in PDAC tissues, we used TISCH2 website to analyze the expression level of SEMA4D in nine public pancreatic cancer datasets, and confirmed that the expression level of SEMA4D was higher in immune cells and lower in cancer cells and stromal cells (Figure 8E).

## Discussion

Despite the success of immunotherapy in various cancers, PDAC remains largely refractory. PDAC is considered as a “cold tumor,” but the mechanisms leading to its development of immune tolerance remain unclear. In this study, we systematically analyzed scRNA-seq from PDAC tumor tissues and PBMC, and the results showed a high proportion of cancer cells and fibroblasts, which is consistent with the fact that PDAC has rich stromal components. However, there are significant differences in cell composition between different samples. Multiple previous studies on the use of scRNA-seq in PDAC have also shown heterogeneity in both tumor epithelial and stromal cells in the microenvironment.^24^^,^^25^^,^^26^ These differences may also be due to the stage of the tumor or the sampling site of the tumor. We found that the proportion of T cells was lower in patients with P1 and P3 (both stage Ⅰb) than in the remaining three patients, indicating that the degree of T cell infiltration in tumor tissue may be correlated with tumor stage. In PBMC, the proportion of myeloid cells was significantly lower in P1 than in the other four patients. Due to sample size limitations, the blood CA19-9 levels of the five patients in this study did not show a correlation between tumor stages. However, the results of a retrospective study of 1,543 patients with pancreatic cancer showed that high serum CA19-9 levels were associated with advanced tumor stage and poor prognosis.^27^ Another study showed that IL-10R2^+^myeloid cells after pancreatectomy indicated tumor recurrence 130 days earlier than CA19-9 positivity.^28^ However, these conclusions still need to be further verified by a large sample size.

We further analyzed the T cells in the dataset, including transcriptomic subtype analysis at single-cell resolution, revealing the characteristics of different subtypes of T cells. The coexistence of multiple subtypes within each tumor also raises the possibility of transcriptomic plasticity between the epithelial and mesenchymal phenotypes that are progressive or responsive to therapy. We found that CD8^+^Tex cells were already present in PDAC tumor tissues at an early stage, suggesting that a robust immunosuppressive microenvironment was formed in the early stage of PDAC. Studies have shown that CD8^+^Tex cells are derived from a pool of Tex cells maintained by stem-like progenitor cells, which can generate terminally differentiated T cells when exposed to prolonged stimulation with tumor antigens.^29^ A study observed the response of CD8^+^T cells to chronic infections in the absence of CD4^+^T cells, and the results showed that the exhausted group of T cells first developed a transitional Teff like subset and eventually progressed to terminal exhaustion.^30^ A similar linear differentiation model was also proposed in a study of high-grade serous ovarian cancer.^31^ In animal models, the progression of T cell exhaustion was confirmed to begin with Tn cells. However, the developmental origin of tumor-reactive T cells in humans may not always be a naive state, with more derived from Tm cells.^32^ TCR sequencing and transcriptional profiling of these tumor-infiltrating T cells showed that the transition from Tcm cells to Tem cells and then to Tex cells is a common trajectory of terminal exhaustion in human cancers.^33^ These results suggest that CD8^+^Teff cells, CD8^+^Tcm cells, and CD8^+^Tem cells are important precursors of CD8^+^Tex cells. In the present study, these three types of cells accounted for a high proportion of tumor tissues, suggesting that CD8^+^Tex cells may be the predominant manifestation of CD8^+^T cells in end-stage PDAC. Evaluation of the origin of tumor-infiltrating T cells revealed the anatomical distribution of different subsets. After antigen-presenting cells capture tumor antigens and carry them to specific regions of lymphoid organs, T cells down-regulate lymphatic homing molecules and up-regulate tissue-infiltrating receptors, allowing them to be transported to the antigen source, where they can differentiate further.^34^ These studies suggest that CD8^+^T cells that differentiate into an exhausted state may be derived from lymphoid tissue and blood circulation. As we found, the proportion of CD8^+^Tex cells in PBMC is very small, but it is mainly dominated by effector cells such as CD8^+^Teff cells and NK cells. However, this study showed that the percentage of CD8^+^Tex cells in PDAC tumor tissues was not high. As studied by Zheng et al., the number of CD8^+^Tex cells was significantly lower in PDAC compared to other digestive system malignancies such as esophageal cancer, liver cancer, cholangiocarcinoma, and colorectal cancer.^33^ This suggests that functional exhaustion of CD8^+^T cells may play a more important role in the immunosuppressive microenvironment of PDAC.

Comparing CD8^+^Tex cells and CD8^+^ non-Tex cells in PDAC tumors, we found that CXCL13 and GZMK were the most significant among the up-regulated and down-regulated DEGs in CD8^+^Tex cells, respectively. The primary function of CXCL13 is to selectively attract B lymphocytes by binding to the BLR1/CXCR5 receptor.^35^ In recent years, CXCL13 has also been found to be closely related to the function of T cells. Yang et al.^36^ demonstrated in both human high-grade serous carcinoma tumors and mouse tumor models that tumor high expression of CXCL13 was able to recruit and activate CXCR5^+^CD8^+^T cells and enhance anti-tumor immunity. However, CXCL13^+^CD8^+^T cells showed an opposite outcome. Li et al.^37^ first reported the role of CXCL13 expression in CD8^+^T cells in melanoma and suggested that CXCL13 is a marker of CD8^+^T cell exhaustion. In this study, we also confirmed that CXCL13 is a biomarker of CD8^+^Tex cells in the PDAC tumor microenvironment. Zheng et al.^38^ produced similar results to ours. A meta-analysis of five cancer types suggested that tumor-specific CXCL13^+^CD8^+^T cells are significantly enriched in tumors that respond to immune checkpoint blockade therapy.^39^ And significantly increased after effective treatment, indicating that tumor-specific CXCL13^+^CD8^+^T cells play a key role in the treatment process. Thus, CXCL13 may be a potential immunotherapy target in PDAC and a key indicator for survival prediction. Granzyme K, the expression product of GZMK, is a serine protease that plays an important role in killing virus-infected cells and cancer cells by NK cells and cytotoxic T lymphocytes.^40^ GZMK^+^T cells express few or no markers associated with T cell exhaustion and are considered to be a group of CD8^+^Tem cells.^41^ Studies have shown that the proportion of GZMK^+^CD8^+^T cells and Tex cells is positively correlated with better survival of patients with non-small cell lung adenocarcinoma and better response of melanoma to immune checkpoint inhibition.^42^^,^^43^ This also proves that GZMK^+^CD8^+^T cells have a function that is diametrically opposite to that of Tex cells.

We found that T cells in the tumor microenvironment of PDAC were closely related to myeloid cells and cancer cells. The close relationship between T cells and myeloid/cancer cells was inferred from both ligand-receptor interaction analysis and spatial proximity in tissue sections. This multi-pronged approach strengthens the conclusion that these cell types are functionally interconnected. Myeloid cells are one of the most abundant cells in the tumor microenvironment, including macrophages, neutrophils, dendritic cells and other types. Tumor-associated macrophages and tumor-associated neutrophils inhibit the recruitment and function of T cells by regulating the secretion of various chemokines and cytokines such as IL-1, IL-10, TGF-β, while tumor-associated dendritic cells inhibit the activation of T cells by reducing the secretion of cytokines such as CXCL9, CXCL9, and IL-7.^44^ Gene mutation and epigenetic recombination in cancer cells are the basis and driving force for their immune escape. Specifically, cancer cells can promote immune escape by directly interfering with the tumor killing ability of T cells or by inducing helper cells in the tumor microenvironment to differentiate into an immunosuppressive phenotype. We found that INHBA^+^macrophages and cancer cells communicate frequently with CD8^+^Tex cells. INHBA is a member of the encoding TGF-β superfamily. Research has shown that upregulating INHBA on macrophages in the tumor microenvironment can promote tumor cell proliferation.^45^ Meanwhile, INHBA^+^macrophages have also been identified as a group of macrophages that upregulate pro-angiogenic signaling pathways.^46^ Sidiropoulos et al. described immune niches associated with immunotherapy response.^47^ Our study also confirmed that INHBA^+^macrophages may affect the occurrence and development of tumors by regulating the exhaustion of CD8^+^T cells. Notably, some INHBA^+^ areas contained abundant CD8A^+^ cells but sparse CD68^+^ cells, indicating that INHBA in those regions was likely derived from non-macrophage sources. Consequently, we focused our spatial analysis on double-positive CD68^+^/INHBA^+^ cells when evaluating potential interactions with CD8^+^ T cells. Future work using multiplex imaging with lineage markers will be required to dissect the precise spatial relationship between CD8^+^ T cells and INHBA^+^ macrophage subtypes.

The prediction of LR interaction analysis also revealed that cell-to-cell interactions in the tumor TME may lead to immune escape. In addition to indirectly regulating CD8^+^T cells by affecting immunosuppressive cells, cancer cells can also directly act on CD8^+^T cells, leading to their functional exhaustion. Based on the analysis and external experimental validation results, we found that SPP1 and integrin α4β1, PLAUR and integrin α4β1 are the key LR pairs for communication between cancer cells and CD8^+^Tex cells. SPP1 is a multifunctional glycoprotein involved in a variety of biological processes, including immune regulation, cell survival and tumor progression.^48^ SPP1 was first thought to be high-expressed in tumor-associated macrophages, causing ‌ their phagocytosis and inflammation to decrease and promoting angiogenesis.^49^ It has also been shown to be related to the exhaustion of CD8^+^T cells and tumor metastasis.^50^^,^^51^ Recently, tumor-derived SPP1 has also been identified as a potent immunosuppressive agent. Tong et al.^52^ demonstrated that cancer cells with high SPP1 expression were preferentially located near hepatic stellate cells, and SPP1 secreted by cancer cells interacted with CD44 receptor on hepatic stellate cells to activate PI3K/AKT signaling pathway and promote the differentiation of hepatic stellate cells into tumor-associated fibroblasts. In addition, tumor-derived SPP1 has also been shown to directly regulate CD8^+^T cells. Klement et al.^53^ suggested that SPP1 expressed by cancer cells inhibits CD8^+^T cell activation and confers host tumor immune tolerance. Song et al.^54^ showed that silencing SPP1 increased the number of CD8^+^T cell infiltrates and the secretion of immunocompetent cytokines in the tumor microenvironment. These results were similar to those in our study. A recent study demonstrated that SPP1 secreted by cancer cells drives mesenchymal cell fate decisions in the PDAC stroma.^55^ Our findings complement this by showing that SPP1 also directly engages with CD8^+^T cells via α4β1 integrin, contributing to T cell exhaustion. Thus, SPP1 may exert dual effects on both stromal and immune compartments, amplifying immunosuppression. PLAUR is a protein expressed on the surface of cancer cells. By binding to urokinase-type plasminogen activator, it promotes the migration, invasion and growth of cancer cells.^56^^,^^57^ In addition, PLAUR activity is also closely related to the immune response in the tumor microenvironment. Studies have shown that PLAUR not only affects tumor cell proliferation and metastasis, but also affects tumor immune evasion and treatment resistance by regulating the function of tumor-associated immune cells.^58^ For example, PLAUR can regulate the immune response in the tumor microenvironment by activating the mitogen-activated protein kinase pathway and affecting the polarization of tumor-associated macrophages. PLAUR can be used as a marker of tumor immunosuppressive characteristics, and samples with high expression of PLAUR have less CD8^+^T cell infiltration and more M2 macrophage infiltration.^59^ PLAUR was also correlated with immune checkpoint expression.^60^ The expression levels of these checkpoint molecules may influence tumor growth and the efficacy of immunotherapy. In this study, we also identified PLAUR as another key molecule involved in CD8^+^T cell exhaustion in PDAC. The protein docking analysis provides structural evidence supporting the ligand-receptor interactions identified by scRNA-seq and spatial transcriptomics. The favorable docking scores for SPP1-α4β1 and PLAUR-α4β1 (−274.80 and −264.10, respectively) indicate that these pairs are not only statistically enriched in LR analysis but also physically plausible; thereby further confirming the credibility of the results. Our findings align with a recent spatial transcriptomics study, which also reported enrichment of exhausted CD8^+^T cells expressing PDCD1, LAG3, and TIGIT in the PDAC stroma. However, our work extends these observations by identifying specific ligand-receptor pairs (SPP1-α4β1, PLAUR-α4β1) that may drive this exhaustion.^61^ In addition, the analysis of multiple datasets also suggests that SPP1 and PLAUR play an important role in the immunosuppressive microenvironment of PDAC, and more evidence will be further confirmed by scientific experimental research in the future.

In summary, by integrating scRNA-seq and ST, we have mapped the landscape of CD8^+^T cell exhaustion in PDAC and uncovered two critical ligand-receptor interactions—SPP1-α4β1 and PLAUR-α4β1—that link cancer cells to exhausted T cells. These interactions are associated with poor prognosis and represent potential targets for reversing immunosuppression. Our study provides a high-resolution resource for understanding T cell dysfunction in PDAC.

### Limitations of the study

However, there are several limitations to this study. First, most of the five PDAC patients were in the early stage, and only one patient was in stage Ⅲ. Therefore, our study may have some limitations in revealing the immunosuppressive microenvironment and CD8^+^ T cell exhaustion status of advanced state PDAC, and only relatively reasonable speculation can be made by combining previous literature reports. Secondly, SPP1 and PLAUR, as two key hub molecules linking PDAC and CD8^+^ Tex cells, may also require more sufficient research evidence, such as *in vivo* and *in vitro* mechanistic verification. Therefore, these basic verification experiments will be the focus of our next research. In our spatial transcriptomics data, SPP1 and PLAUR expression appeared largely non-overlapping in some tumor regions, which may reflect intratumoral heterogeneity. However, complementary evidence from multiplex immunofluorescence, protein docking, and survival analyses supports the biological relevance of both SPP1-α4β1 and PLAUR-α4β1 interactions with CD8^+^ Tex cells. A detailed region-based assessment of CD8^+^ T cell infiltration and exhaustion according to SPP1/PLAUR status would require high-plex spatial phenotyping and is therefore a direction for future investigation.

## Resource availability

### Lead contact

Requests for further information and resources should be directed to and will be fulfilled by the lead contact, Qiang Yan ( yianq@hzhospital.com).

### Materials availability

This study did not generate new unique reagents.

### Data and code availability

• Sequencing data (scRNA-seq and spatial transcriptomics) have been deposited in the Gene Expression Omnibus (GEO) under accession number GSE335452.
• All custom code used for data processing and analysis is available from the lead contact upon request.
• Any additional information required to reanalyze the data reported in this article is available from the lead contact upon request.

## Acknowledgments

This work is supported by 10.13039/100014718National Natural Science Foundation of China (82273339), Key research and development project of 10.13039/501100008990Science and Technology Department of Zhejiang Province (2024C03174), 10.13039/501100010248Public Welfare Technology Application Research Program of Huzhou (2021GZ73), 2023 Zhejiang Province Special Support Program for High-Level Talents: Healthcare Leading Talents Project and Zhejiang Province First Batch of “Small yet Strong” Clinical Innovation Team Project.

## Author contributions

J.M.: writing – review and editing, writing – original draft, visualization, software, methodology, investigation, formal analysis, data curation, and conceptualization; C.Y.: writing – review and editing, methodology, investigation, formal analysis, and conceptualization; Y.M.: investigation and visualization; Y.Y.: data curation and conceptualization; X.Y.: investigation and conceptualization; J.Z.: methodology and validation; K.Y.: investigation and conceptualization; G.G.: software and data curation; H.Z.: methodology and visualization; Y.Z.: investigation and visualization; Y.W.: visualization and data curation; S.H.: methodology, validation, project administration, and conceptualization; Q.Y.: validation, project administration, funding acquisition, and conceptualization.

## Declaration of interests

The authors declare no competing interests.

## STAR★Methods

### Key resources table

**Table:** 

REAGENT or RESOURCE	SOURCE	IDENTIFIER
**Antibodies**		

Anti-pan Cytokeratin antibody	Abcam	Cat# ab80826; RRID: AB_1640401
Anti-PD1 antibody	Abcam	Cat# ab237728; RRID: AB_3073606
Anti-CD8 alpha antibody	Abcam	Cat# ab316778; RRID: AB_3107047
Anti-Semaphorin 4D/CD100 antibody	Abcam	Cat Num: ab307685
Anti-CD45 antibody (PTPRC)	Abcam	Cat Num: ab318154
Anti-uPA Receptor/U-PAR antibody	Abcam	Cat# ab221680; RRID: AB_3662121
Anti-Integrin beta 1 antibody	Abcam	Cat# ab52971; RRID: AB_870695
Anti-VISTA antibody	Abcam	Cat# ab300042; RRID: AB_3696949
Anti-HLA E antibody	Abcam	Cat# ab300553; RRID: AB_2943209
Anti-Osteopontin antibody (SPP1)	Abcam	Cat# ab214050; RRID: AB_2894860
Anti-Inhibin beta A antibody	Abcam	Cat# ab97705; RRID: AB_10680598
Anti-CD68 antibody	Abcam	Cat# ab283654; RRID: AB_2922954

**Software and algorithms**		

R	R Foundation	https://www.r-project.org/
GraphPad Prism	GraphPad Software	Version 8.0

**Deposited data**		

TCGA	NCI	https://www.cancer.gov/tcga
GEO	NCBI	https://www.ncbi.nlm.nih.gov/geo/

### Experimental model and study participant details

#### Collection of clinical human samples

This study has been approved by the Medical Ethics Committee of Huzhou Hospital, Zhejiang University School of Medicine (202112031-01). Five fresh tissue samples and matched fresh blood samples were obtained from patients who underwent surgical resection of pancreatic cancer without neoadjuvant therapy at the Department of Hepatobiliary and Pancreatic Surgery, affiliated Huzhou Hospital, Zhejiang University School of Medicine. Blood samples were taken before surgery. Clinical information such as age, gender, pathological characteristics, and preoperative serum CA19-9 level were collected. Written informed consent was obtained from all participants prior to sample collection.

### Method details

#### Tissue dissociation and single-cell suspensions preparation

Fresh PDAC tissues were placed in sterile RNase-free dishes containing ice-cold calcium- and magnesium-free 1× PBS. Tissues were minced into approximately 0.5 mm^2^ pieces, washed with PBS, and carefully dissected to remove blood clots and adipose tissue. Dissociation was performed in enzyme solution at 37°C with shaking at 100 rpm for 20 min. Digestion was stopped by adding 1× PBS supplemented with 10% fetal bovine serum, followed by gentle pipetting 5–10 times. The suspension was passed through a 70 μm cell strainer and centrifuged at 300×g for 5 min at 4°C. The cell pellet was resuspended in 100 μL of 1× PBS containing 0.04% BSA, mixed with 1 mL of 1× red blood cell lysis buffer, and incubated at room temperature or on ice for 2–10 min to eliminate erythrocytes. After centrifugation (300×g, 5 min, room temperature), dead cells were removed using the Miltenyi Dead Cell Removal Kit according to the manufacturer’s instructions. The resulting single-cell suspension was washed twice with 1× PBS/0.04% BSA, resuspended in 50 μL of the same buffer, and assessed for viability by trypan blue exclusion. Only suspensions with >85% viability were used; cell counts were determined using a Countess II Automated Cell Counter, and concentrations were adjusted to 700–1200 cells/μL.

#### ScRNA-seq procedure

Single-cell suspensions were loaded onto the 10× Genomics Chromium platform to generate gel beads in emulsion using the Chromium Single-Cell 3′Reagent Kit (v3) following the manufacturer’s protocol. Subsequent cDNA amplification and library construction were performed according to standard procedures. Libraries were sequenced on an Illumina NovaSeq 6000 system with paired-end 150 bp reads, targeting a minimum depth of 20,000 reads per cell. Sequencing was carried out by LC-Bio Technology Co., Ltd. (Hangzhou, China).

#### Quality control and data processing

Raw sequencing data were demultiplexed and converted to FASTQ format using Illumina bcl2fastq (version 2.20). We used the 10× Genomics official analysis software Cell Ranger (version 3.1.0) to perform quality control on the raw sequencing data of each sample and to align reads to the reference genome (GRCh38). The Cell Ranger output was loaded into Seurat (version 3.1.(1) for dimensionality reduction, clustering, and analysis of scRNA-seq data. Quality control thresholds were set based on standard practices in scRNA-seq analysis. Cells with fewer than 500 detected genes were excluded as they likely represent empty droplets or damaged cells. Cells with more than 5,000 detected genes were considered potential doublets or multiplets and were also removed. The mitochondrial gene expression threshold was set at <25%, as a high percentage of mitochondrial reads is a common indicator of cell stress or compromised membrane integrity.

#### Dimension reduction and clustering

To visualize the data, we further reduced the dimensionality of the filtered cells using Seurat and used UMAP to project the cells into two-dimensional space. Using the LogNormalize method of the “Normalization” function of the Seurat software to calculated the expression value of genes. Principal component analysis (PCA) was used to reduce the dimension and reduce the variables, and then the homogenized expression values were used for PCA analysis. The first 10 principal components were selected from the results of PCA analysis for subsequent clustering and grouping analyses. Cell clusters were identified by clustering algorithm optimized based on shared nearest neighbor module. Marker genes for each cluster were identified with the Wilcoxon rank-sum test (default parameters is “bimod”: Likelihood-ratio test) with default parameters via the FindAllMarkers function in Seurat. The annotated marker genes for each large class of cells are detailed in Table S1, and the annotated marker genes for T cell subclasses refer to previous studies reported.^62^ Marker genes were selected if they were expressed in more than 10% of cells in a cluster and had an average log2 fold change >0.25.

#### Differential expression and enrichment analysis

Differentially expressed genes (DEGs) between CD8^+^Tex and non-Tex cells were identified with thresholds: |log2FC| >0.25, expression in >25% of cells in the target cluster, and adjusted *p* < 0.05. Visual volcano map was drawn according to the differential gene results. The Symbol identifier of differential genes was converted into Entrez ID by clusterProfiler R package (version 4.0), and all genes detected by experiments were used as background gene set to eliminate technical bias. GO enrichment analysis covered three ontologies: Biological Process (BP), Molecular Function (MF) and Cellular Component (CC). Correction for multiple hypothesis testing was performed with the use of the Benjamini-Hochberg procedure (significance threshold: adjusted *p* < 0.05). KEGG pathway analysis aimed at species-specific metabolic and regulatory pathways, and the latest pathway annotation was obtained through KEGG REST API. The enrichment results were visualized by enrichplot package (version 1.20.0). Significantly enriched entries (top 20) were independently manually reviewed, and the localization of genes in the Pathway map was verified by the KEGG Pathway database (https://www.kegg.jp/).

#### Cell–cell interaction analysis

We used the “CellPhoneDB” R package to perform high-resolution characterization analysis of cell-cell interactions. The potential interaction strength between cells was calculated using the “CellPhoneDB” R package according to the official workflow. It visualizes the detailed interaction patterns and each ligand-receptor (LR) pair and draws a visual heatmap based on the number of LR relationship pairs. We quantified cell-cell communication based on the number of significant LR pairs and their average expression levels in interacting cell types.

#### ST data analysis

ST was performed on five FFPE PDAC sections using the 10× Genomics Visium Spatial platform. Raw sequencing data were processed with Space Ranger (version 1.1). Gene point matrices generated after processing data from ST and Visium samples were analyzed using the Seurat software package (version 3.1.1). Genes with a minimum number of detected genes of 200 were screened, and genes with fewer than 10 readings or expression below 3 points were removed. The different spots were normalized using the LogVMR function. After dimensionality reduction and clustering, the SpatialFeaturePlot function in Seurat (version 1.1.(1) was used to generate spatial feature expression maps.

#### Multiplex immunofluorescence staining

FFPE sections were subjected to multiplex immunofluorescence staining following standard protocols. After dewaxing, antigen retrieval, and blocking, sections were incubated with primary antibodies (listed below) overnight at 4°C, followed by HRP-conjugated secondary antibodies and TSA fluorophore development. Nuclei were counterstained with DAPI. Images were acquired using a fluorescence microscope. The primary antibodies used included CK (1:2000, Abcam, ab80826), PD-1 (1:2500, Abcam, ab237728), CD8 (1:500, Abcam, ab316778), SEMA4D (1:1000, Abcam, ab307685), PTPRC (1:10000, Abcam, ab318154), plasminogen activator urokinase receptor (PLAUR) (1:500, Abcam, ab221680), α4β1 (1:250, Abcam, ab52971), VSIR (1:500, Abcam, ab300042), HLA-E (1:500, Abcam, ab300553), Secreted phosphoprotein 1‌ (SPP1) (1:10000, Abcam, ab214050), INHBA (1:1000, Abcam, ab97705), CD68 (1:2000, Abcam, ab303565).

#### Spatial docking of proteins

Since the exact three-dimensional structure of integrin α4β1 is still unknown, the amino acid sequences of Integrin alpha-4 and integrin beta-1 were obtained from UniProt (https://www.uniprot.org/). Then the sequence of import Swiss-model (https://swissmodel.expasy.org/interactive) in the prediction of α4β1 protein structure. The Ramachandran plot was used to evaluate the rationality of the predicted protein structure. After downloading the SPP1 (PDB ID: 3DSF) and PLAUR (PDB ID: 2FD6) protein structures from the RCSB PDB website (https://www.rcsb.org/), α4β1 and SPP1, α4β1 and PLAUR were docked using the HDOCK (http://hdock.phys.hust.edu.cn/) online service platform and visualized in PyMOL (version 2.3.0).

#### Public database analysis

Using TIMER2.0 (http://timer.cistrome.org/), we analyzed the correlation between key tumor genes and CD8^+^T cell infiltration. We used CIBERSORTx (available in TIMER2.0) as it estimates absolute cell fractions and has been shown to perform well in solid tumors. The GEPIA2 database (http://gepia2.cancer-pku.cn/index.html) was used to compare the expression levels of key molecules between normal pancreas (from GTEx) and PDAC tumors (from TCGA_PAAD), as well as across different tumor stages. In Kaplan-Meier Plotter website (https://kmplot.com/analysis/) using the RNA-seq data of the carcinoma in the TCGA key tumor gene prognosis for survival curve drawing. The expression levels of key molecules in various types of cells in tumor tissues were analyzed on TISCH2 database (http://tisch.comp-genomics.org/).

### Quantification and statistical analysis

All statistical analyses were performed with the R tool (version 4.2.2). The K-M method and the corresponding log rank test were used to determine the prognostic value of the selected genes. Independent sample *t* test and Mann-Whitney U test were used to compare means between groups for continuous variables. Pearson/Spearman test was used for correlation analysis. Statistical significance was defined as ∗*p* < 0.05.

## Supplemental information

Document S1. Figures S1–S3

Table S1. Marker genes for the annotation of cell types in PDAC

Table S2. Clinical and pathological features of five patients with PDAC

Table S3. The number of cells used for single-cell transcriptome sequencing

Table S4. Top ten marker genes in each cell cluster of tumor tissue in PDAC

Table S5. Top ten marker genes in each cluster of PBMC in PDAC

Table S6. The number of T cell subsets used for single-cell transcriptome sequencing

Table S7. Top ten marker genes of various T cells in tumor tissues of PDAC

Table S8. Top ten marker genes of various T cells in PBMC of PDAC

Table S9. DEGs between CD8_Tex cells and CD8_other cells in tumor tissues

Table S10. DEGs between CD8_Tex cells and CD8_other cells in PBMC

Table S11. Mean expression of significant results for each receptor-ligand pair in cell-to-cell interaction analysis
