# Supplementary material for: Single-cell RNA-seq and spatial transcriptomics characterize CD8+ exhausted T cells in pancreatic ductal adenocarcinoma
Source: iScience. 2026 Jul 27;29(8):116934. doi: 10.1016/j.isci.2026.116934 (PMC13429902; doi:10.1016/j.isci.2026.116934)
Supplement: Document S1. Figures S1–S3 [file mmc1.pdf]

iScience, Volume *29*

Supplemental information

Single-cell RNA-seq and spatial transcriptomics characterize $CD8^+$ exhausted T cells in pancreatic ductal adenocarcinoma

Jing Mao, Chenxin Yan, Ying Mei, Yanjun Yao, Xingxia Yang, Jing Zhuang, Kuai Yu, Gangzhao Gu, Hengzhi Zhang, Yu Zheng, Yunyao Wei, Shuwen Han, and Qiang Yan

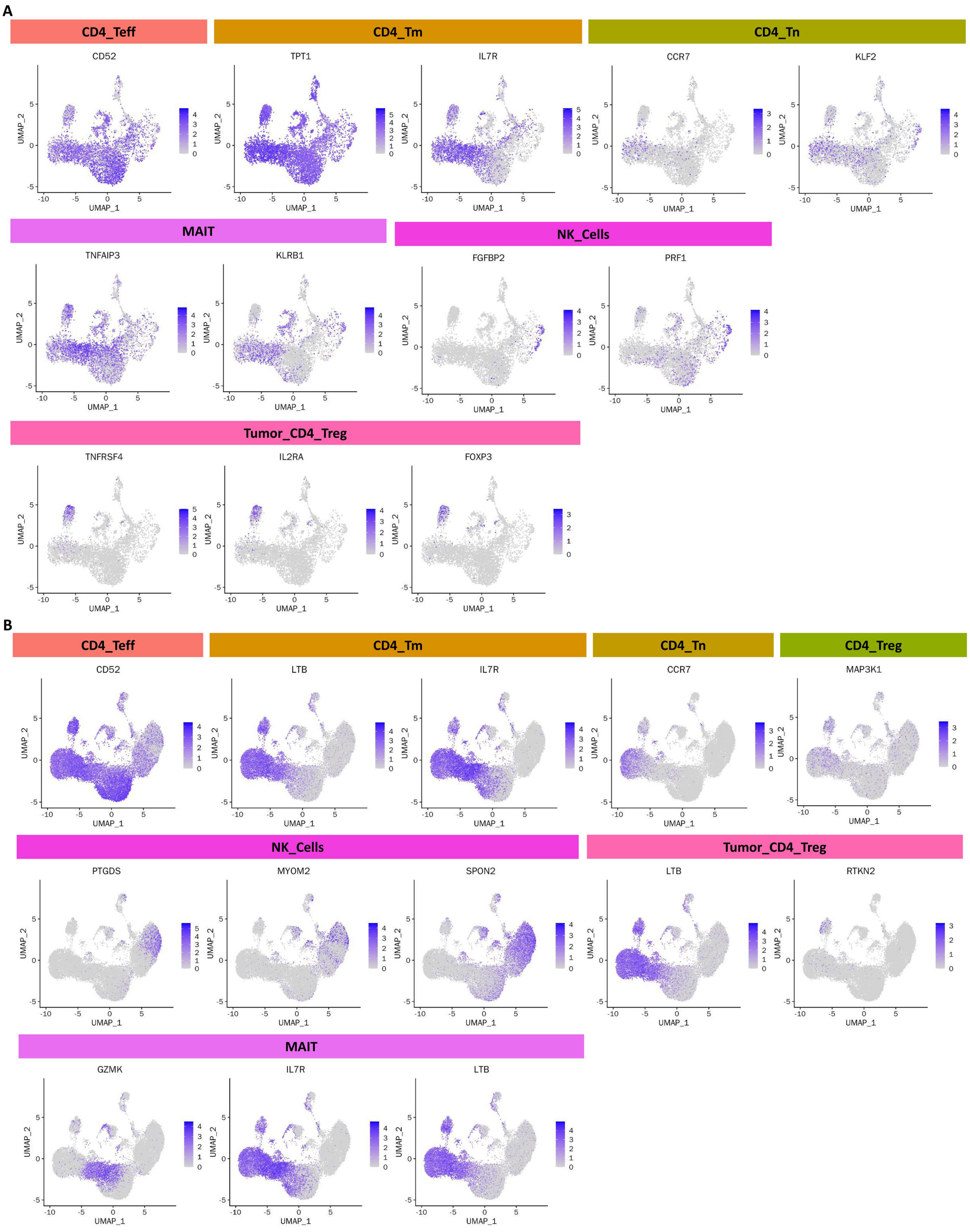

Supplementary Figure 1. Molecular markers of in CD8^{+}T cells in PDAC tumor tissues and PBMC.

(A) Expression levels of the selected marker genes across 6,715 T cells in UMAP maps from tumor tissues in PDAC patients. (B) Expression levels of the selected marker genes across 24,427 T cells in UMAP plots from PBMC in PDAC patients.

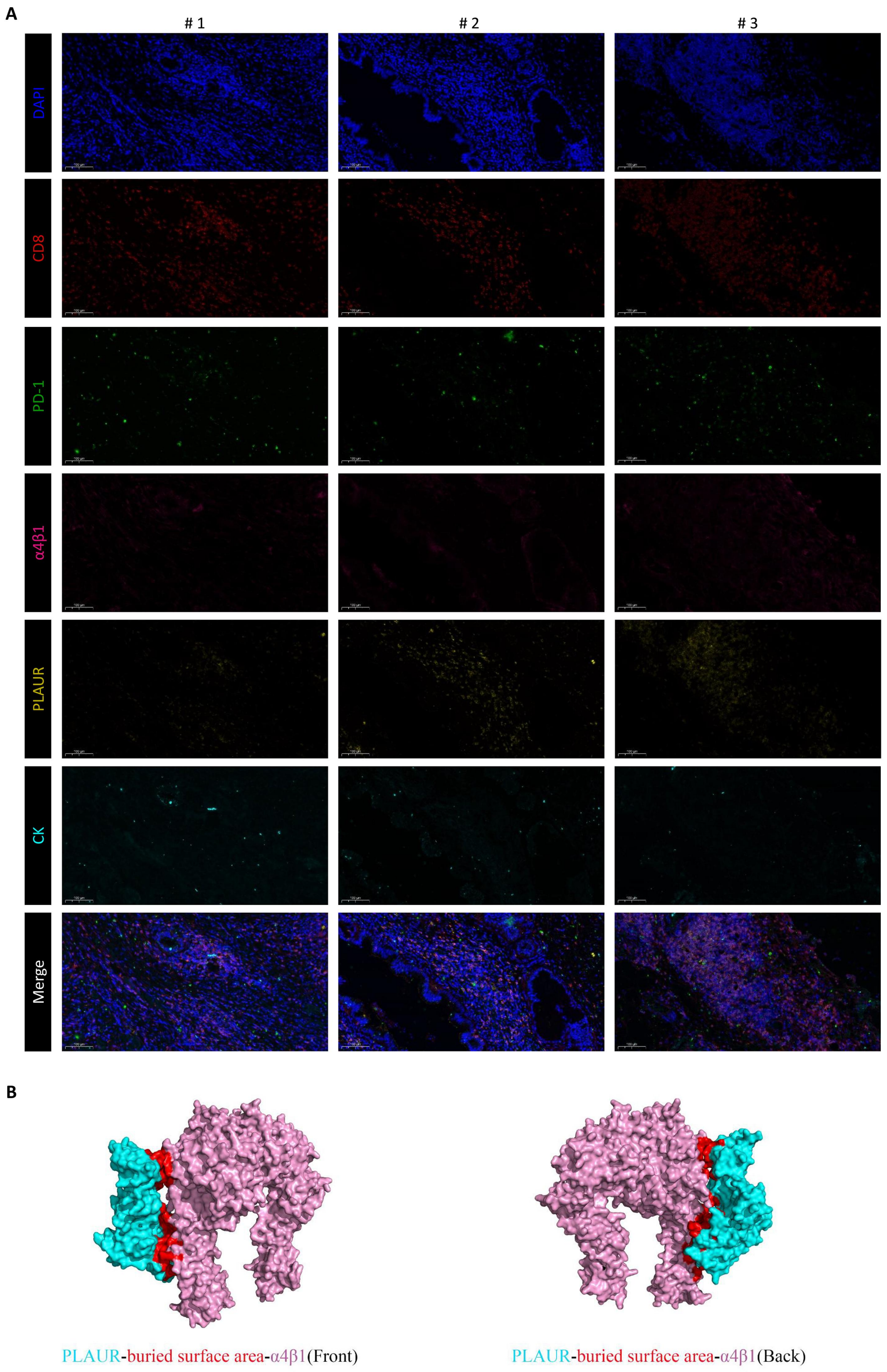

Supplementary Figure 2. Relationship between PLAUR$^+$cancer cells and α4β1$^+$CD8$^+$Tex cells in PDAC.

(A) Multiplex immunofluorescence staining of human PDAC tumor tissue (20×). CK (cyan), DAPI (blue), CD8 (red), PD-1 (green), α4β1(pink), and PLAUR (orange), in individual and merged channels are shown. Bar, 100 μm. The experiments were performed in five patients.

(B) Visualization analysis by PyMOL showing that α4β1(purple) and PLAUR (cyan) have good spatial binding (front and back). The binding site is shown in red.

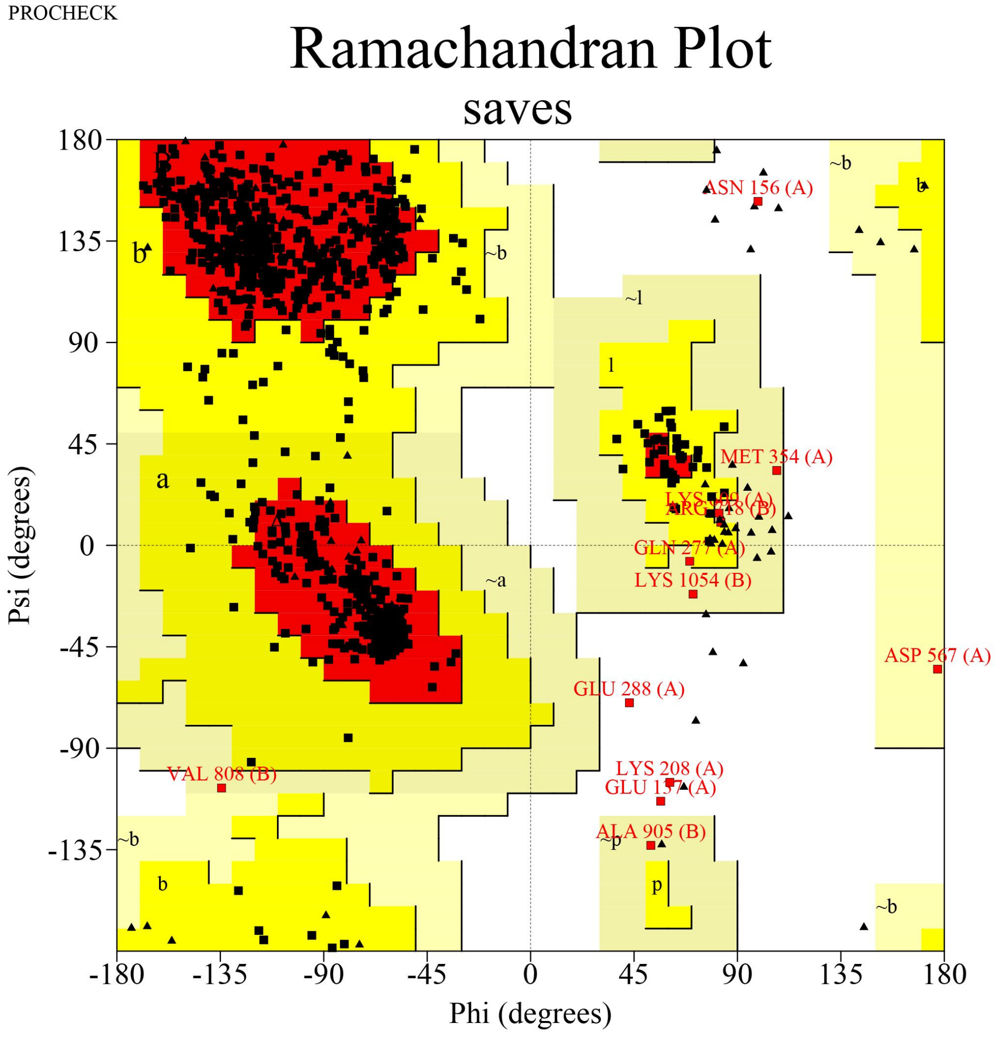

Plot statistics

Residues in most favoured regions [A,B,L]	786	88.5%
Residues in additional allowed regions [a,b,l,p]	90	10.1%
Residues in generously allowed regions [~a,~b,~l,~p]	8	0.9%
Residues in disallowed regions	4	0.5%
	----	------
Number of non-glycine and non-proline residues	888	100.0%
Number of end-residues (excl. Gly and Pro)	7	
Number of glycine residues (shown as triangles)	89	
Number of proline residues	46	

Total number of residues	1030	

Based on an analysis of 118 structures of resolution of at least 2.0 Angstroms and R-factor no greater than 20%, a good quality model would be expected to have over 90% in the most favoured regions.

Supplementary Figure 3. Ramachandran plot for evaluating the rationality of α4β1 predicted structure.

Amino acid residues that fall in the most favoured regions and the additional allowed regions account for more than 90% of the whole protein.
